# Bibliographical

**Published:** 1891-03

**Authors:** 


### Bibliographical.

BY B. H. CATCHING, D.D.S., EDITOR AND PUBLISHER, ATLANTA, GA.

Catching's Compendium of Practical Dentistry for 1890.

This volume is selections of valuable practical methods and processes, from the dental journalistic literature of the past year, and as a work of ready reference for the busy practitioner is very valuable indeed. The average practitioner does not take all the journals, indeed very few take more than from one to three, and perhaps in these instances they are too often but hastily scanned so that many good things are liable to escape attention.

The aim of Dr. Catching has been to cull from all the dental journals, these practical matters and put them in such form and arrangement, that they may be available, with the least possible inconvenience, to the practitioner, he has succeeded well, when it is considered that it was a first effort in this line. Nothing of the kind had ever been attempted by any one before.

This work should have a place in every dentist's office and certain it is, that wherever it may be, it will be often consulted.

It is to be hoped that the doctor will meet such encouragement that he will prepare and issue such a work each year.

We hope his most ardent anticipations will be fully met, and that he will continue in the good work he has so well begun.

